# Postoperative Unstimulated Thyroglobulin Accurately Predicts Outcomes in High‐Risk Differentiated Thyroid Cancer: A Retrospective Cohort Study

**DOI:** 10.1111/cen.15260

**Published:** 2025-05-04

**Authors:** Julia Isabel Richter Cicogna, Sophia Yada Noguchi, Adriano Namo Cury, Giovanna Marcela Vieira Della Negra, Laís de Oliveira Teles Fraga, Marcelo Soares Schalch, Rafael de Cicco, Carolina Ferraz da Silva, Rosália Do Prado Padovani

**Affiliations:** ^1^ Faculdade de Ciências Médicas da Santa Casa de São Paulo (Faculty of Medical Sciences of Santa Casa de São Paulo) São Paulo Brazil; ^2^ Irmandade da Santa Casa de Misericórdia de São Paulo São Paulo Brazil; ^3^ Instituto do Câncer Doutor Arnaldo Vieira de Carvalho Sao Paulo Brazil

**Keywords:** prognosis, thyroglobulin, thyroid cancers

## Abstract

**Objective:**

High‐risk differentiated thyroid cancer (DTC) patients show variable outcomes. While postoperative stimulated thyroglobulin (STg) is a recognized predictive marker, the prognostic significance of unstimulated thyroglobulin (UTg) is still unexplored. This study aims to assess the prognostic value of postoperative UTg in high‐risk DTC patients.

**Design:**

Retrospective cohort study (2015–2024) at two Brazilian tertiary hospitals.

**Patients:**

One thousand DTC patients were identified, of which 144 were high‐risk. Fifty seven patients met the inclusion criteria.

**Methods:**

Clinical, pathological, and laboratory data were collected. Outcomes were categorized as favorable (excellent/indeterminate responses) or unfavorable (biochemical/structural incomplete responses). Receiver Operating Characteristic (ROC) curves determined cutoff values for predicting outcomes and metastases.

**Results:**

Significant predictors of unfavorable outcomes included advanced age (*p* = 0.048), larger tumor size (*p* = 0.002), higher UTg (*p* < 0.001), and STg (*p* < 0.001). UTg was an independent risk factor for 1‐year outcomes (OR = 0.008; 95% CI: 0.001–0.088; *p* < 0.001). UTg cutoff of 2.1 ng/mL distinguished outcomes with high sensitivity (83.3%), specificity (96.0%), and accuracy (90.7%). A higher cutoff of 3.8 ng/mL identified metastases (sensitivity 86.4%, specificity 90.5%). UTg showed non‐inferiority to stimulated thyroglobulin (STg) in predicting outcomes (*p* = 0.964) and metastasis (*p* = 0.980).

**Conclusion:**

Postoperative UTg is a strong prognostic marker in high‐risk DTC patients, providing a non‐inferior alternative to STg with greater accessibility and fewer side effects. We propose a clinical algorithm to optimize the management of these cases. When UTg levels exceed 2.1 ng/mL, particularly higher than 3.8 ng/mL, investigation of potentially resectable metastatic foci should be considered before radioiodine therapy. Prospective studies are needed to validate this algorithm.

## 1 Introduction

Differentiated thyroid cancer (DTC) generally has an excellent prognosis, with a 5‐year survival rate of about 98.5% in the United States [1]. However, up to 15% of cases are diagnosed with local invasion [2], and the incidence of advanced cases and mortality directly attributed to DTC has risen [3]. These trends highlight the necessity of identifying patients at risk for developing advanced disease to optimize treatment strategies.

The American Joint Committee on Cancer—AJCC/TNM staging system [4] categorizes DTC into stages based on tumor, node, and metastasis (TNM) criteria. While stage I patients over 55 years have a 10‐year survival rate of 98%–100%, stage IV patients experience significantly poorer outcomes, with survival rates below 50% [5]. Various factors not considered by this staging system—including specific histological types, thyroglobulin (Tg) levels, molecular profiles, and metastases’ location and functional status [6, 7, 8]—significantly impact prognosis, emphasizing the importance of investigating these variables.

The American Thyroid Association (ATA) risk stratification system [9] is a widely used tool to predict recurrence. However, 10%–30% of high‐risk patients achieve excellent outcomes within 5–10 years [10], highlighting the heterogeneity of this group. Identifying high‐risk patients likely to respond favorably could reduce overtreatment and its associated risks, such as adverse effects of high‐dose RIT and TSH suppression therapy.

Tg is a highly specific tumor marker in DTC management [11]. Its serum levels reflect thyroid tissue mass and TSH receptor stimulation [11]. Tg can be measured during TSH suppression therapy with levothyroxine (LT4) or following TSH stimulation to increase diagnostic sensitivity [11]. However, recombinant TSH therapy, which avoids the need for LT4 withdrawal, remains cost‐prohibitive in many countries and is not currently available in the Brazilian public health system (SUS) [12]. LT4 withdrawal reduces quality of life due to the onset of hypothyroidism symptoms.

Postoperative Tg levels before radioiodine therapy (RIT) correlate with residual disease, metastasis, and recurrence risk [13]. While postoperative stimulated Tg (STg) is widely studied and values below 10 ng/mL are seen as reassuring [9], the role of postoperative unstimulated Tg (UTg) remains underexplored, particularly in high‐risk patients. Advances in Tg assays with functional sensitivity as low as 0.1–0.2 ng/mL now allow accurate measurements [14], reducing the need for TSH stimulation [15, 16, 17] and potentially streamlining prognostic evaluations.

Although STg values < 1 ng/mL are associated with excellent responses in high‐risk patients [18], the utility of postoperative UTg in this context has not been investigated. This study aims to assess postoperative UTg as a prognostic marker in high‐risk DTC patients, correlating its values with 1‐year treatment responses and identifying precise cutoff points for clinical use.

## 2 Materials and Methods

### 2.1 Study Design and Data Collection

This retrospective cohort study evaluated medical records of thyroid cancer patients treated at two tertiary hospitals in São Paulo, Irmandade da Santa Casa de Misericordia de São Paulo (ISCMSP) and Instituto do Câncer Doutor Arnaldo (IAVC), Brazil, between 2015 and 2024. Data collection included clinical, pathological, ultrasonographic, and laboratory information obtained during diagnosis, treatment, and 1‐year follow‐up. Study data were managed using the REDCap (Research Electronic Data Capture) system [19]. The research was conducted according to the guidelines of STROBE (Strengthening the Reporting of Observational Studies in Epidemiology) [20]. The study adhered to ethical guidelines and was approved by the ethics committees of both institutions under the Certificate of Ethical Appreciation numbers 73496023.8.1001.5479 and 73496023.8.2001.5471. All patients attending medical consultations during the data collection period signed the informed consent form.

### 2.2 Study Population

Among 1487 records reviewed, 1000 cases of DTC were identified, with 144 (14.4%) classified as high‐risk for recurrence or persistence according to ATA guidelines [9]. After applying exclusion criteria, 57 high‐risk patients were included in the final analysis. Exclusion criteria comprised: anti‐thyroglobulin antibody positivity (*n* = 28, 19.4%; defined by detectable levels per institutional assay cutoffs), non‐DTC neoplasms on histopathology review (*n* = 1), incomplete histopathological documentation (*n* = 12), treatment that deviated from ATA recommendations for high‐risk DTC [9] (*n* = 1), a follow‐up period of less than 1 year after total thyroidectomy and RIT (*n* = 7), and incomplete or sparse medical records, (*n* = 38). No study candidates declined participation.

### 2.3 Laboratory Methodology

Laboratory analyses of UTg and STg were performed employing the Elecsys Tg II kit [21], from Roche Diagnostics, Germany, via electrochemiluminescence, with an analytical sensitivity of 0.04 ng/mL, and functional sensitivity of 0.1 ng/Ml [22]. Tests conducted in external laboratories were included if they adhered to CRM 457 standardization [23]. Postoperative UTg levels were measured under TSH suppression (TSH < 2 mIU/L, preferably < 0.1 mIU/L), ideally 30 days after total thyroidectomy and before RIT. STg levels were measured after TSH stimulation (TSH > 30 mIU/L) before RIT.

### 2.4 Statistical Analysis

Qualitative variables were presented as frequencies and percentages, while quantitative data were summarized as means with standard deviations for normal distributions or medians with interquartile ranges for non‐normal distributions. Chi‐square or Fisher's exact tests were employed to compare categorical variables, whereas Student's *T*‐test or Mann–Whitney *U* test was utilized for continuous variables. Multivariate logistic regression was implemented to identify independent risk factors. Receiver Operating Characteristic (ROC) curves were created to establish optimal Tg cutoff values for predicting treatment responses and metastases. Treatment outcomes were categorized as favorable (excellent and indeterminate responses) or unfavorable (biochemical and structural incomplete responses). For metastatic disease, outcomes were classified as presence (structural incomplete response) or absence (all other responses). Sensitivity, specificity, positive predictive value (PPV), and negative predictive value (NPV) were calculated for each cutoff. The Youden Index (YI) was employed to find the optimal cut‐off. A p‐value of less than 0.05 was deemed statistically significant. All analyses were conducted using SPSS software (version 20, SPSS Inc., Chicago USA).

## 3 Results

### 3.1 Patient's Characteristics

Among the 57 high‐risk differentiated thyroid cancer (DTC) patients included in the study, 71.9% were female, and the median age at diagnosis was 49.6 years. Most patients (91.2%) had papillary thyroid carcinoma, predominantly the classic subtype (45.6%), while 8.8% had follicular carcinoma. Approximately 22.8% had distant metastases at diagnosis, with the lungs being the most affected site (19.3%). All patients received radioiodine therapy (RIT) following total thyroidectomy, with a mean administered dose of 200 mCi. Serum thyroglobulin levels were measured at least 4 weeks after surgery (median of 62 days post‐thyroidectomy) and before RIT. After 1 year of follow‐up, 31.6% of patients achieved an excellent response, while 54.4% were classified as having an incomplete structural response. The remaining patients showed indeterminate (5.3%) or biochemical incomplete responses (8.8%). The patient's characteristics are detailed in Table 1.

**Table 1:** Clinical and histopathological characteristics of patients.

**Female, n(%)** a	41 (71.9%)
**Age, years** b	49.6 ± 16.2
**BMI (kg/m** ^**2**^ **)** c	27.5 [24.2–31.8]
**Comorbidities, n (%)** a	
Obesity	17 (29.8%)
Overweight	17 (29.8%)
Smoking	13 (22.8%)
Personal history of other cancer	5 (8.8%)
Family history of thyroid cancer	6 (10.5%)
**Total thyroidectomy, n (%)** a	
One stage	55 (96.5%)
Two stages	2 (3.5%)
**Lymph node dissection, n (%)** a	37 (64.9%)
Central lymph node dissectionα	25 (43.9%)
Lateral lymph node dissectionβ	16 (28.1%)
**Excision of extrathyroidal extension** γ, **n (%)** a	16 (28.1%)
**Tumor size, (cm)** c	3 [2‐ 6]
**Histological subtypes, n (%)** a	3 [2–6]
Papillary carcinoma	52 (91.2%)
Classic	26 (45.6%)
Follicular	12 (21.1%)
Follicular carcinoma	5 (8.8%)
**Additional findings in anatomopathological exam, n (%)** a	
Multifocality	25 (43.9%)
Gross ETE^3^	22 (38.6%)
Lymph node metastasis	39 (68.4%)
**TNM staging, n (%)** a	
T3/T4	39 (68.4%)
N1	41 (71.9%)
M1	13 (22.8%)
**Location of distant metastasis, n (%)** a	
Lungs	11 (19.3%)
Mediastinum	2 (3.5%)
Spine	1 (1.8%)
Other bones	5 (8.8%)
**AJCC/TNM staging** [4], **n (%)** a	
< 55 years, stage II:	30 (52.6%)
> 55 years, stage III and IV:	15 (26.3%)
**Postoperative status**	
Unstimulated Tg (ng/mL)^c^	4 [0.4–174.2]
Tg essay performed by the institution's laboratory, n (%)^a^	28 (49.1%)
Time since surgery (days)^c^	62 [36–90]
**Radioiodine Theraphy, dose (mCi)** c	200 [150–250]
Stimulated Tg (ng/mL)^c^:	36.3 [5.7–307]
**Response to treatment, n (%)** a	
Excellent	18 (31.6%)
Indeterminate	3 (5.3%)
Biochemical incomplete	5 (8.8%)
Structural incomplete	31 (54.4%)
**Follow‐up time (years)** c	1.3 [1–1.15]
**Total, n**	57

Abbreviations: %, percentage; cm, centimeters; mCi, millicuries; ng/mL, nanograms per milliliter; TNM, tumor‐node‐metastases, staging system of the American Joint Commission on Cancer (AJCC) [6].

^a^ Frequencies and percentages for categorical variables;

^b^ Mean ± standard deviation;

^c^ Median [interquartile ranges].

^α^ Lymph node dissection of central chains (anatomical levels VI and VII);

^β^ Lymph node dissection of lateral chains (levels II to V);

^γ^ Excision of gross Extrathyroidal Extension (ETE): tumor invading into the strap muscles, subcutaneous tissue, trachea, or other organs, visible during surgery or on naked‐eye examination.

### 3.2 Risk Factors

In univariate analysis, significant predictors of unfavorable outcomes included older age at diagnosis (*p* = 0.048), larger tumor size on histopathology (*p* = 0.002), and higher levels of both postoperative UTg (*p* < 0.001) and STg (*p* < 0.001). Patients with unfavorable outcomes had a median UTg of 61.8 ng/mL and STg of 235 ng/mL, compared to 0.4 and 5 ng/mL, respectively, in those with favorable outcomes, as specified in Table 2. In multivariate logistic regression, only UTg was an independent risk factor for 1‐year outcomes, with an odds ratio of 0.008 [95% Confidence Interval (CI): 0.001–0.088; *p* < 0.001].

**Table 2:** Comparative analysis of clinical and anatomopathological characteristics of high‐risk DTC patients with outcomes in the first year of follow‐up.

Characteristics	Response to treatment		*p* value	Test
	Favorable 21 (36.8%)	Unfavorable 36 (63.2%)		
Age (years)^b^	44 ± 15.6	52.8 ± 15.9	0.048*	Student *T* test
Female sex^a^	16 (76.2%)	25 (69.4%)	0.585	Chi‐square
Obesity^a^	6 (28.6%)	10 (27.8%)	0.949	Chi‐square
Overweight^a^	6 (28.6%)	12 (33.3%)	0.709	Chi‐square
BMI (kg/m^2^)^c^	28.6 [1.3–3.5]	27.1 [24.8–31.8]	0.811	Mann‐Whitney
Smoking^a^	4 (19.0%)	9 (25.0%)	0.748	Fisher's exact
Personal history of cancer^a^	0 (0%)	5 (13.9%)	0.146	Fisher's exact
Family history of CDT^a^	3 (14.3%)	3 (8.3%)	0.659	Fisher's exact
Tumor size (cm)^c^	2.2 [1.3–3.5]	4.5 [2.5–7.6]	0.002*	Mann–Whitney
Papillary carcinoma^a^	21(100%)	31 (86.1%)	0.146	Fisher's exact
Follicular carcinoma^a^	0 (0%)	5 (13.9%)	0.146	Fisher's exact
Papillary classical subtipe^a^	9 (42.9%)	17 (47.2%)	0.663	Chi‐square
Papillary folicular subtipe^a^	7(33.3%)	5 (13.9%)	0.092	Fisher's exact
Capsular invasion^a^	4 (19.0%)	10 (27.8%)	0.460	Chi‐square
Gross ETE^a^	6 (28.6%)	16 (44.4%)	0.235	Chi‐square
AJCC/TNM T3/T4^a^	12 (57.14%)	27 (75.0%)	0.115	Chi‐square
N1a or N1b^a^	17 (81.0%)	24 (66.7%)	0.311	Chi‐square
M1^a^	0 (0%)	13 (36.1%)	0.001*	Fisher's exact
< 55 years, stage II^a^	0 (0%)	5 (13.9%)	0.045*	Fisher's exact
> 55 years, stage III ou IV^a^	3 (5.3%)	12 (33.3%)	1.000	Fisher's exact
Unstimulated Tg (ng/ml)^c^	0.4 [0.1–1.1]	61.8 [8.4–367]	< 0.001*	Mann–Whitney
Stimulated Tg (ng/ml)^c^	5 [1.2–26.2]	235 [16.2–819.2]	< 0.001*	Mann–Whitney

Abbreviations: %, percentage; BMI, Body Mass Index; cm, centimeters; DTC, Differentiated Thyroid Carcinoma; mCi, millicuries; ng/mL, nanograms per milliliter; TNM, tumor‐node‐metastases, staging system of the American Joint Commission on Cancer (AJCC) [6].

^a^ Frequencies and percentages for categorical variables;

^b^ Mean ± standard deviation;

^c^ Median [interquartile ranges]. Significance: *p* < 0.05. The variables with *p* ≤ 0.10 in the univariate analysis were evaluated subsequently by the multivariate analysis.

### 3.3 UTg and STg Cutoffs and Diagnostic Performance Metrics

**Figure 1:**
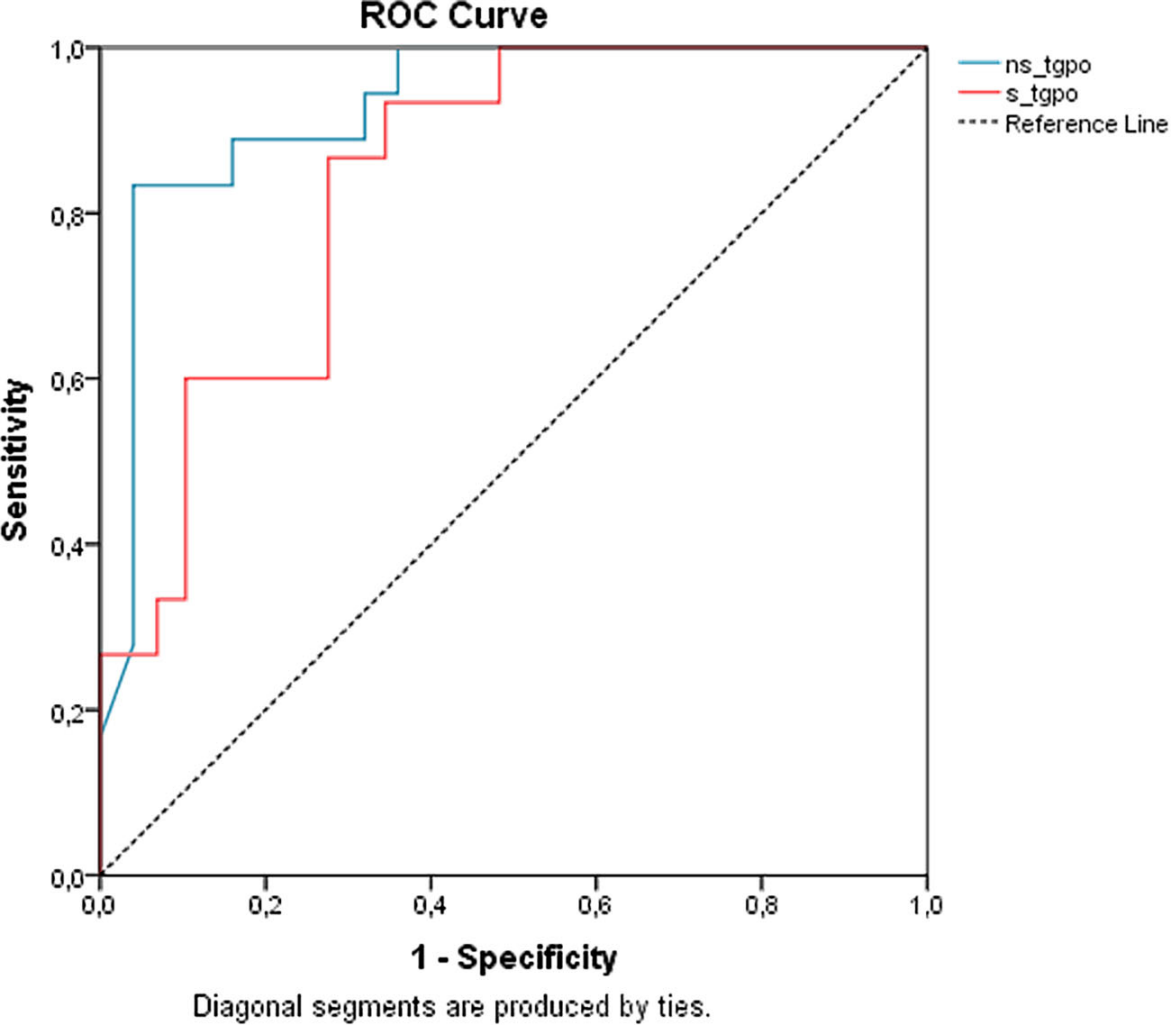
ROC Curve Comparing Unstimulated and Stimulated Thyroglobulin Tests in Distinguishing Favorable and Unfavorable Responses in 1 Year. *Note:* ROC curve: Receiver Operating Characteristic. ns_tgpo: Unstimulated thyroglobulin (*p* < 0.001, AUC: 0.929, CI: 0.850–1.000). s_tgpo: Stimulated thyroglobulin (*p* < 0.001, AUC: 0.839, CI: 0.724–0.954). Favorable response: excellent and indeterminate responses. Unfavorable response: biochemical and structural incomplete responses.

ROC curve analysis demonstrated the prognostic accuracy of UTg and STg. For distinguishing favorable from unfavorable outcomes, the UTg cutoff of 2.1 ng/mL showed a sensitivity of 83.3%, specificity of 96.0%, and accuracy of 90.7%, with an area under the curve (AUC) of 0.929 (95% CI: 0.850–1.000; *p* < 0.001), and a Youden Index (YI) of 0.793. The STg cutoff of 32.6 ng/mL demonstrated a sensitivity of 86.7%, specificity of 72.4%, and accuracy of 77.3%, with an AUC of 0.839 (95% CI: 0.724–0.954, *p* < 0.001), and YI of 0.591. These results are detailed in Table 3 and Figure 1. Formal comparison using *Z*‐test revealed no statistically significant difference between these AUCs (*p* = 0.964), despite UTg's numerically higher value.

**Table 3:** Analysis of postoperative UTg and STg cutoff values in predicting favorable or unfavorable treatment responses at one year of follow‐up in high‐risk DTC patients.

Markers	Cutoff values			
	UTg 2,1 ng/mL		STg 32,6 ng/mL	
	Estimate	95% CI	Estimate	95% CI
Sensitivity	83.3%	58.6%–96.4%	86.7%	59.5%–98.3%
Specificity	96.0%	79.7%–99.9%	72.4%	52.8%–87.3%
PPP	93.8%	68.5%–99.0%	61.9%	46.6%–75.2%
NPP	88.9%	74.0%–95.8%	91.3%	73.9%–97.5%
Accuracy	90.7%	77.9%–97.4%	77.3%	62.2%–88.5%

*Note:* Favorable response: excellent and indeterminate responses. Unfavorable response: biochemical and structural incomplete responses.

Abbreviations: CI, confidence interval; NPP, negative predictive value; PPP, positive predictive value; STg, stimulated thyroglobulin; UTg, unstimulated thyroglobulin.

**Figure 2:**
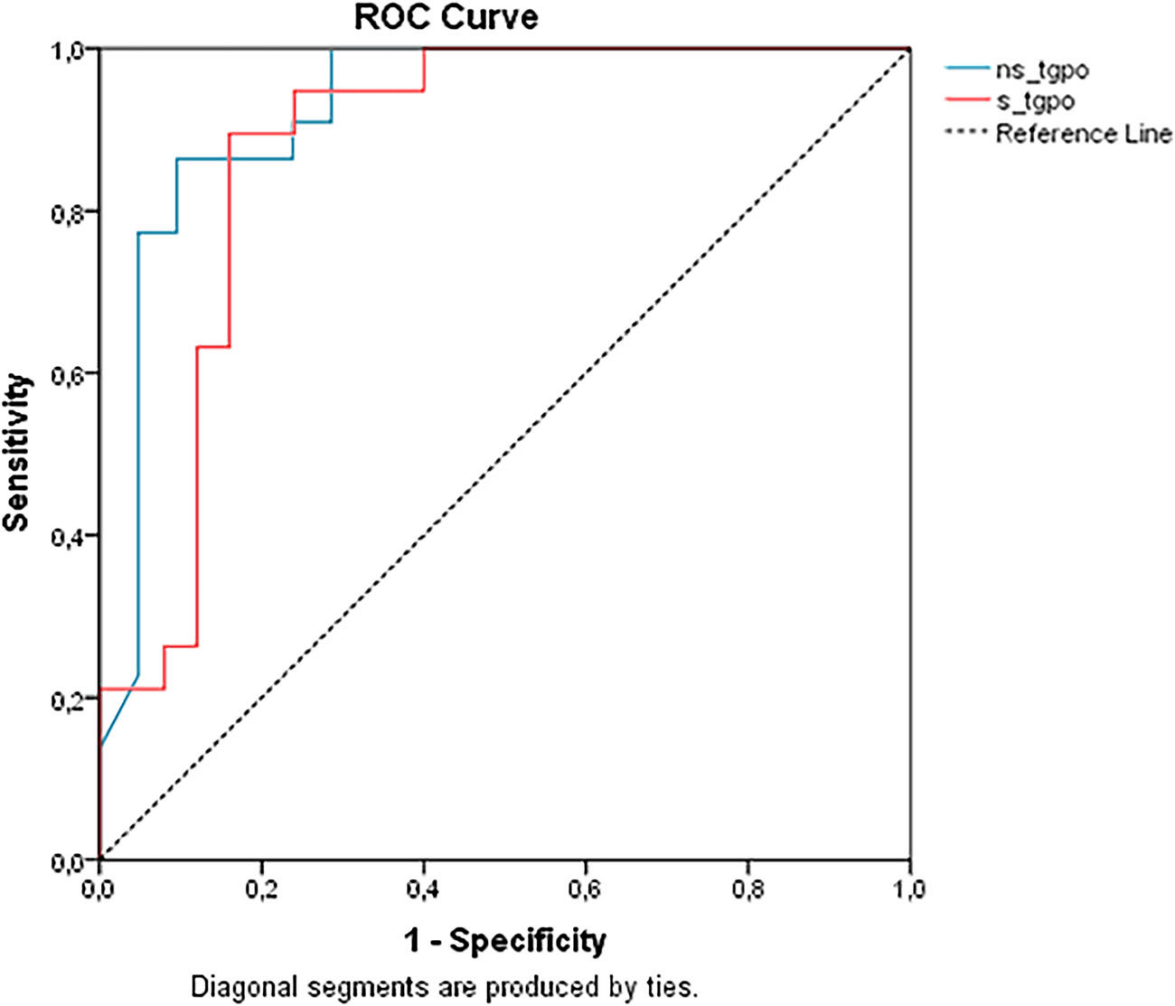
ROC Curve Comparing Unstimulated and Stimulated Thyroglobulin Tests in Distinguishing between the presence or absence of metastasis in the first year of follow‐up. *Note:* ns_tgpo: Unstimulated Tg (*p* < 0.001, AUC 0.926, 95% CI: 0.841 – 1.000); s_tgpo: Stimulated Tg (*p* < 0.001, AUC: 0.876, 95% CI: 0.768–0.984).

For predicting metastases, a UTg cutoff of 3.8 ng/mL achieved sensitivity of 86.4%, specificity of 90.5%, and accuracy of 88.4%, with an AUC of 0.926 (CI: 0.841–1.000; *p* < 0.001) and YI of 0,768, while the STg cutoff achieved sensitivity of 89.5%, specificity of 84.0%, and accuracy of 86.4%, with an AUC of 0.876 (95% CI: 0.768–0.984; *p* < 0.001) and YI 0,735, as displayed in Table 4 and Figure 2. Despite UTg's numerically higher AUC, direct comparison using the *Z*‐test showed no significant difference between the biomarkers (*p* = 0.980), consistent with their overlapping confidence intervals. These results indicate comparable prognostic utility for metastatic prediction.

**Table 4:** Analysis of postoperative UTg and STg cutoff values in predicting metastasis at one year of follow‐up in high‐risk DTC patients.

Markers	Cutoff values			
	UTg 3.8 ng/mL		STg 32.6 ng/mL	
	Estimate	95% CI	Estimate	95% CI
Sensitivity	86.4%	65.1%–97.1%	89.5%	66.9%–98.7%
Specificity	90.5%	69.6%–98.8%	84.0%	63.9%–95.5%
PPP	90.5%	71.6%–97.3%	81.0%	63.1%–91.4%
NPP	86.4%	68.7%–94.8%	91.3%	73.7%–97.5%
Accuracy	88.4%	74.9%–96.1%	86.4%	72.7%–94.8%

Abbreviations: CI, confidence interval; NPP, negative predictive value; PPP, positive predictive value; STg, stimulated thyroglobulin; UTg, unstimulated thyroglobulin.

## 4 Discussion

This study demonstrates that postoperative unstimulated thyroglobulin (UTg) is a reliable and independent prognostic marker for predicting 1‐year treatment responses in high‐risk DTC patients. UTg and STg showed statistically comparable performances, with UTg exhibiting a pattern of numerically higher AUCs across both outcome prediction and metastatic risk assessment. These findings highlight the clinical value of UTg in identifying high‐risk patients who may benefit from more intensive follow‐up or treatment.

Higher UTg levels were strongly associated with poorer outcomes, as patients with biochemical or structural incomplete responses had significantly elevated UTg compared to those with excellent or indeterminate responses. A UTg cutoff of 2.1 ng/mL demonstrated excellent sensitivity (83.3%), specificity (96.0%), and accuracy (90.7%) for predicting treatment responses, while a cutoff of 3.8 ng/mL effectively identified patients at risk of metastases. Notably, UTg demonstrated better specificity and fewer false positives than STg, making it a valuable alternative, especially in situations where TSH stimulation is not feasible or accessible.

**Figure 3:**
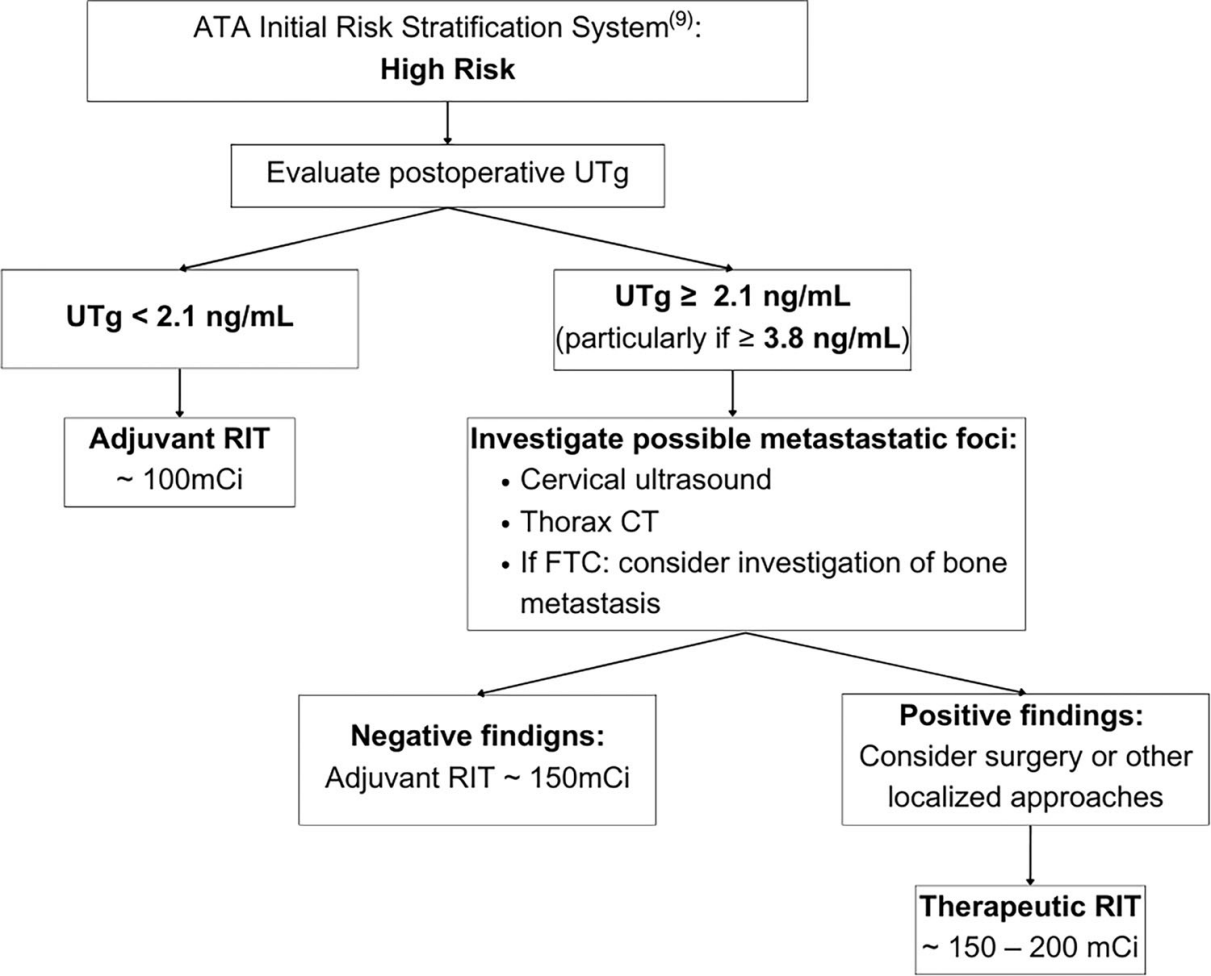
Proposed clinical algorithm for postoperative evaluation of high‐risk DTC patients. *Note:* CT, computed tomography; FTC, follicular thyroid carcinoma; mCi, millicuries; RIT, radioiodine therapy; UTg, unstimulated thyroglobulin.

Based on our findings, we propose a UTg‐guided clinical algorithm (Figure 3) to optimize postoperative management of high‐risk DTC patients. Patients with postoperative UTg levels > 2.1 ng/mL—particularly those exceeding 3.8 ng/mL—should undergo comprehensive metastatic evaluation (e.g., cervical ultrasound, thoracic CT). If metastatic disease is confirmed, we recommend surgical resection or localized therapies before radioiodine therapy (RIT) to maximize therapeutic efficacy. This approach not only individualizes RIT dosing (100 mCi for adjuvant use vs. 150–200 mCi for therapeutic intent) but also reduces reliance on stimulated testing. Prospective studies are warranted to validate this algorithm's impact on clinical outcomes and refine implementation protocols.

This study findings are consistent with previous studies on the prognostic role of STg [24] but expand current knowledge by establishing UTg as a similarly effective marker. Studies have shown that postoperative STg values between 10 and 30 ng/mL are associated with an increased risk of recurrence and persistence [9]. In the present work, the STg cutoff of 32.6 ng/mL had the highest accuracy for distinguishing outcomes, consistent with studies that found similar values, such as Yang et al. [25], who reported a cutoff of 26.75 ng/mL, and Kelly et al. [26], who found a value of 30 ng/mL for predicting a poorer treatment response.

Few studies have evaluated the prognostic significance of postoperative UTg. Ibrahimpasic et al. [27] in 2012 demonstrated that low‐ to intermediate‐risk patients with a postoperative UTg of < 1 ng/mL experienced excellent clinical outcomes and recurrence rates of less than 1%; however, the functional sensitivity of the method used was 1 ng/mL. More recently, in 2019, McDow et al. [28] reported that none of the 66 low/intermediate‐risk CPT patients with postoperative UTg ≤ 0.2 ng/mL developed structural recurrence in a 6‐month follow‐up. To our knowledge, this study is the first to evaluate the prognostic role of postoperative UTg in high‐risk DTC patients and to find highly accurate predictive cutoff values.

This study adds to the evidence by showing that UTg can reliably predict outcomes while providing several practical advantages. UTg measurements eliminate the hypothyroid symptoms related to levothyroxine withdrawal and the high costs and limited availability of recombinant TSH, especially in resource‐limited settings. Advanced age, larger tumor size, and higher Tg levels were identified as significant predictors of unfavorable outcomes, consistent with prior research [29, 30].

Although some studies have compared stimulated and unstimulated thyroglobulin in DTC follow‐up [31, 32], no articles have compared these variables obtained postoperatively and before radioiodine therapy. In the present study, it was found that UTg has a comparable capacity to STg in distinguishing treatment responses among high‐risk patients who may show either favorable or unfavorable responses after 1 year (AUC: 0.929 and 0.839, respectively) and in the presence or absence of metastases (AUC: 0.926 and 0.876, respectively), with no statistical difference. However, UTg demonstrated numerically higher AUCs across both outcome prediction and metastatic risk assessment than STg, suggesting potential clinical advantages worthy of further investigation. Additionally, UTg served as an independent risk factor for predicting outcomes at 1 year, establishing it as a relevant biomarker for disease progression.

The STg cutoff value exhibited high sensitivity (86.7% and 89.5%, respectively, for favorable or unfavorable outcomes and the presence or absence of metastases) and a high negative predictive value (91.3% in both cases). These results indicate that this marker effectively identifies patients with unfavorable outcomes or metastases after 1 year of follow‐up when values exceed the established cutoff (32.6 ng/mL). However, its specificity (72.4% and 84%, respectively) is lower, resulting in a higher rate of false positives than UTg. Therefore, caution in interpreting its results may be necessary due to the potential for unnecessary interventions in cases of false positives.

Despite the necessity for TSH stimulation in high‐risk DTC patients for RIT, these findings suggest that the reliability of postoperative UTg is high, allowing for prognostication as early as the first month of follow‐up. Our experience shows that many patients treated through the Brazilian Unified Health System (SUS) face challenges accessing RIT. Consequently, prompt prognostication can help prioritize patients for necessary treatment. Another practical application of these findings is to guide the frequency of examinations and medical follow‐up visits. However, further studies are required to substantiate these hypotheses as formal recommendations.

Historically, total thyroidectomy and RIT were standard procedures for all patients with differentiated thyroid carcinoma (DTC) [33]. With the expansion of knowledge surrounding this disease, refinement of risk stratification methods, and, more recently, an understanding of the molecular mechanisms involved in DTC pathophysiology, a more individualized approach has been adopted, avoiding unnecessary procedures that may result in side effects [33].

McDow et al. [28] suggest that postoperative non‐stimulated ultra‐sensitive thyroglobulin (UTg) should guide the decision regarding the administering of RIT in low‐ and intermediate‐risk DTC patients. In high‐risk patients, while current evidence indicates that all should receive RIT [9], it is crucial to recognize that the high‐risk group is heterogeneous, with some patients exhibiting excellent responses—31.65% of cases in this study showed excellent responses within 1 year, corroborated by the literature [18, 19]—and considering that treatment with 131I carries potential side effects, we recommend that further studies investigate whether postoperative UTg, in conjunction with imaging examinations, can guide the prescribed dose of 131I to avoid unnecessary overdosing.

The high positive predictive value of the cutoff values identified for UTg indicates that a positive result is reliable for identifying patients with a higher likelihood of poor treatment responses. This can support clinical decisions to enact more rigorous interventions in patients at greater risk of recurrence, potentially enhancing disease management. A study conducted in Japan [34] found no significant difference between the treatment with a low iodine dose (approximately 30 mCi) and high doses (approximately 80–100 mCi) in patients with intermediate and high‐risk DTC, excluding those with distant metastases at diagnosis. A pre‐RIT UTg cutoff of 4 ng/mL was identified as a significant factor for treatment failure. Further studies are necessary to test these hypotheses.

Despite its strengths, this study has limitations. The study's relatively small sample size and substantial exclusion rate may introduce selection bias, a limitation we acknowledge. This primarily reflects the inherent constraints of retrospective design. While this limitation is shared by similar retrospective studies in this field, we mitigated potential bias by applying uniform exclusion criteria and providing detailed documentation of excluded cases (Section 2.2). The retrospective design and reliance on medical records may have introduced information bias, and the lack of standardized measurements across external laboratories could have affected results. The study's follow‐up period was limited to 1 year [35]. Although this timeframe is a strong predictor of long‐term outcomes, longer follow‐up is necessary to confirm these findings and assess the utility of UTg in guiding treatment decisions.

Our study intentionally focuses on high‐risk DTC patients—a challenging subgroup that represents only 10%–15% of all DTC cases [9] but accounts for a disproportionate share of disease‐specific morbidity and mortality, particularly where evidence supporting the use of unstimulated thyroglobulin (UTg) is scarce. We believe that the insights gained from studying this targeted group provide valuable data that may not be captured in larger, more heterogeneous populations. While the number of patients in our study is indeed small, the focus on this rare and challenging cohort allows for a deeper understanding of their unique clinical characteristics and treatment responses, which can inform future research and clinical practice.

## 5 Conclusion

Postoperative unstimulated thyroglobulin is a highly accurate and accessible biomarker for predicting treatment responses in high‐risk DTC patients. UTg demonstrated non‐inferiority to STg and presented practical advantages, including ease of measurement, fewer side effects, and wider availability. A UTg cutoff of 2.1 ng/mL effectively identified patients at risk for unfavorable outcomes, while a cutoff of 3.8 ng/mL distinguished those likely to develop metastases. Based on our findings, we propose a clinical algorithm to optimize postoperative management of high‐risk DTC patients. We recommend incorporating routine postoperative UTg measurements for high‐risk patients. When UTg levels exceed 2.1 ng/mL, especially higher than 3.8 ng/mL, further investigation of potentially resectable metastatic foci should be considered before radioiodine therapy. Prospective studies are needed to validate this clinical algorithm and to establish clear guidelines for its implementation.

## Conflicts of Interest

The authors declare no conflicts of interest.
